# The interaction of high-power fiber laser irradiation with intrusive rocks

**DOI:** 10.1038/s41598-021-04575-z

**Published:** 2022-01-13

**Authors:** Youngjin Seo, Dongkyoung Lee, Sukhoon Pyo

**Affiliations:** 1 Department of Future Convergence Engineering, Kongju National University, Cheonan, 31080 South Korea; 2 Department of Mechanical and Automotive Engineering, Kongju National University, Cheonan, 31080 South Korea; 3 Department of Urban and Environmental Engineering, Ulsan National Institute of Science and Technology (UNIST), Ulsan, 44919 South Korea

**Keywords:** Engineering, Materials science

## Abstract

Laser cutting of intrusive rocks, including granite, gabbro, and diorite, is carried out in order to assess the cut characteristics through geometrical measurements, such as kerf width, melting width, and penetration depth. The absorption rate for each specimen at the wavelength of 1064 nm is measured using a spectrophotometer. A multimode fiber laser is used in this study with the power of 9 kW and different cutting speeds. Furthermore, nitrogen gas at 13 bar is applied as the assistant gas in order to remove the melted material effectively. As a result of the experiment, the relationship between the cutting speed and geometrical measurements is investigated. Furthermore, variations of penetration depth are performed in accordance with the number of laser cuts. In addition, through energy dispersive X-ray (EDX) element mapping, minerals that comprise the rocks are classified and characterized. Subsequently, the changes in the microstructure and chemical composition of each specimen, before and after laser cutting, are compared using scanning electron microscope (SEM) and EDX analyses. Experimental results demonstrate that the cutting characteristics vary, depending on the types of minerals that make up the rock. Based on a series of tests, it is identified that volume energy of more than 3.06E + 13 $\mathrm{J}/{\mathrm{m}}^{3}$ is required to fully cut intrusive rocks that have a thickness of 25 mm.

## Introduction

Intrusive rocks account for more than 90% of the Earth’s crust and constitute one type of igneous rocks. They are formed through the solidification of magma that is typically derived from the Earth’s mantle. Depending on the silicon dioxide (${\mathrm{SiO}}_{2}$) content of the bulk rock composition, intrusive rocks can further be classified into three categories: granite, gabbro, and diorite. These rocks include various minerals and are ideally suited for application in construction, civil engineering, landscaping, and monumental spheres because of their aesthetic appeal and high resistance, which allows them to withstand adverse weather conditions due to their high material stability and durability.

Conventional rock cutting is usually performed by employing diamond coated tools, e.g., diamond blades^1^ or diamond saw wires^2^. The performance of these tools relies on the characteristics of the cutting system and of the rock being cut. The cutting quality and the wear rate correlate to the hardness of the rock. Furthermore, high noise and dust levels are generated during the cutting process. Some of the fine dust particles produced by the rock cutting process can harm the health of rock manufacturing workers. These facts, above all, make the rock cutting process an arduous and hazardous task, one that is significantly unsafe for workers. In addition, the vibrations made by the cutting machine cause the creation of macro- and micro-cracks, which could result in unnecessary rock defects near the machining area.

To overcome these problems, abrasive water-jet cutting is used as an alternative to diamond saw cutting^3,4^. Arab et al.^5^ investigated the influences of abrasive water-jet cutting and demonstrated the effects of mechanical properties on the material removal mechanism of abrasive water-jet cutting. Wang et al.^6^ evaluated the accuracy of the numerical simulation results through experiments examining how a particle water-jet impacts the rock. The influence of a particle water-jet on the evolution of damage and the rock failure process were studied using smooth particle hydrodynamics (SPH) together with the finite element method (FEM)—that is, the combined SPH-FEM method. This process cuts rocks more effectively in comparison to diamond cutting but has disadvantages, such as a high noise level, a high kerf taper angle, and a tendency to break the workpiece down during water-jet processing.

The laser-aided manufacturing (LAM) process, especially laser cutting, can be applied to rocks without the drawbacks of the aforementioned mechanical cutting methods. LAM has many advantages—e.g., it is a non-contact method that has low noise and dust levels, ease of automation, and high precision. Furthermore, since LAM has a very small heat-affected zone (HAZ), there is very little material deformation resulting from heat. Due to these superiorities, LAM has been applied in a wide range of industry fields despite the fact that it has higher investment costs than conventional cutting^7–11^.

Several attempts to study the applicability of lasers on rocks are found in the literature^12–14^. Erfan et al.^15^ conducted the Nd: YAG laser perforation of different rocks, such as granite, limestone, and shale. They developed a prediction formula in accordance with laser average power, using the rate of perforation (ROP) and specific energy (SE) values. In addition, they reported that the applicability of moving perforation and a precise cylindrical hole can be created by equalizing the ROP and laser head speed. Yan et al.^16^ proposed an interaction mechanism between laser and sandstone using a 10 kW fiber laser perforation. Laser power and irradiation time were explained as important variables of laser perforation on sandstone. Li et al.^17^ evaluated the influence of laser processing parameters on the characteristics of laser perforation. Based on the microstructural characteristics found via the scanning electron microscope (SEM) and the crack distributions that occur around the laser perforation, fracture mechanisms and physical processes were offered.

As mentioned above, most studies found in the literature explained the possibility of laser-based processing of rocks and related materials. Nonetheless, there is not much data in terms of systemizing the effect of laser parameters on the maximum processing rates and quality criteria during the laser cutting process of various types of rocks. Furthermore, the physical and chemical variations in rocks after laser irradiation have not yet been considered in detail and the correlations between the number of laser cuts and the penetration depth of rocks have not been studied.

Hence, this paper aims to propose the applicability of laser cutting as an alternative to mechanical rock cutting techniques. The specimens used in this study comprised three types of intrusive rocks: granite, gabbro, and diorite. First, the absorption rate for each specimen was measured using a spectrophotometer. Then, cutting experiments were conducted using a high-power multimode fiber laser. Following the experiments, geometrical measurements—such as kerf width, melting width, and penetration depth—were observed. In addition, the relationship between the number of laser cuts and penetration depth was established. Finally, the changes in the microstructure and chemical composition, both before and after laser irradiation, were analyzed using SEM and energy dispersive X-ray (EDX).

## Experimental procedure

### Specimens

The base materials used in this study were commercially available intrusive rocks: granite, gabbro, and diorite. Granite collected at Pocheon city (Gyeonggi-do) is the most common intrusive rock, with silicon dioxide comprising more than 68% of its weight^18^. It is a coarse-grained intrusive rock that is primarily composed of quartz, k-feldspar, plagioclase, and biotite. Since the granite has a high mechanical strength, it is used for civil engineering structure that requires flooring and load support among external construction materials. Gabbro is a silica-poor plutonic rock that is chemically equivalent to basalt. It normally has a coarse-grained texture and typically contains minerals such as k-feldspar, pyroxene, and olivine. Diorite has similar texture to granite; however, their compositional mineral types differ. It is primarily composed of plagioclase and minerals that contain high amount of metallic element such as iron and magnesium. All tested materials have the same dimensions: $100\mathrm{ mm}\times 100\mathrm{ mm}\times 25\mathrm{ mm}$ (width $\times$ length $\times$ height).

Table 1 shows the compressive strength, specific gravity, and water absorption rate for each rock. The granite has the highest compressive strength and water absorption among rock specimens used in this experiment. The specimens covered by a plastic sheet were stored at room temperature to perform the water absorption test. Values of water absorption for each rock are calculated by the weight variation as follows^19^:

1 $$W=\frac{{M}_{w}-{M}_{d}}{{M}_{d}} \times 100\%,$$

where $W(\%)$ is the water absorption of rock specimen, ${M}_{w}$ and ${M}_{d}$ are the weight after immersion of specimen in water for 48 h and the weight of the dry state, respectively. The water absorption is determined by the water holding capacity of the rocks. High water absorption causes the mechanical disruption of an externally exposed surface of rock and facilitates the degree and speed of weathering into the rock. In addition, high water absorption affects to the internal microcrack and volume-expansion in rocks allowing for higher porosity. In general, water absorption and specific gravity are inversely proportional regardless of rock type. In order to measure the density of rocks, it is necessary to measure the volume. However, it is difficult to do accurately. Thus, in geology, specific gravity is used instead of the density. Specific gravity is defined as the ratio between the mass of rock to the mass of the same volume of water. This parameter can help to identify the rock type and their geologic structure. Most of rocks in the earth have specific gravity less than 3.0. Besides, the rock having specific gravity of more than 2.55 can be applied for heavy construction work^20^.

**Table 1:** Values of compressive strength, specific gravity and water absorption for the studied rocks.

Rock type	Compressive strength $[\mathrm{kg}/{\mathrm{cm}}^{2}]$	Specific gravity	Water absorption $[\mathrm{\%}]$
Granite	1990	2.59	0.39
Gabbro	1549	2.85	0.14
Diorite	1870	2.68	0.28

**Figure 1:**
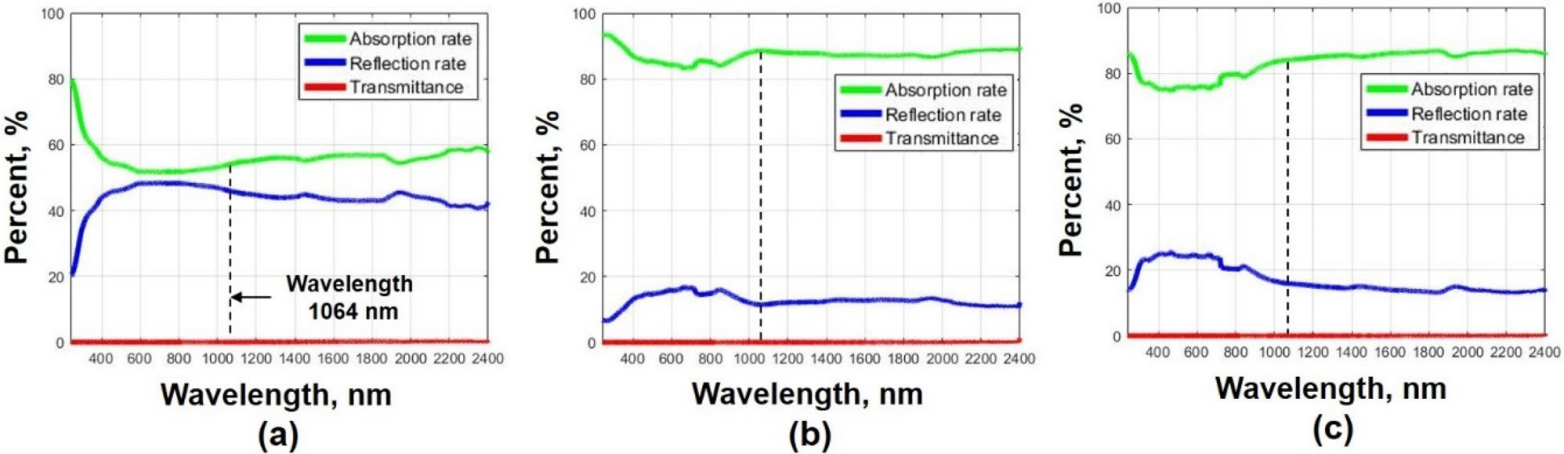
Comparison of the absorption rates of (**a**) granite, (**b**) gabbro, and (**c**) diorite at various wavelengths.

Before performing laser cutting on intrusive rocks, the absorption rate of each specimen was measured. Although the absorption rate of the material is one of the most important properties in laser processing, the studies that measure the absorption rate of rocks are very rare. Therefore, to address this research gap, the absorption rate of each specimen was measured using UV–VIS-NIR spectrophotometers (SolidSpec-3700, Shimadzu, Kyoto, Japan) at wavelengths ranging from 200 to 2400 nm. The results are summarized in Fig. 1. Since the laser used in the experiments had a wavelength of 1064 nm, the absorption rate was measured at this wavelength. The absorption rates at the wavelength of 1064 nm for granite, gabbro, and diorite were 54.4%, 88.7%, and 83.1%, respectively. Granite had the lowest absorption rate among the specimens. This might be explained by the fact that granite had the highest weight percentage of silicon dioxide. Consequently, it was observed that the reflectivity of granite was the highest among the specimens. Meanwhile, gabbro had the highest absorption rate due to its low silicon dioxide content. Based on these results, the influence of silicon dioxide content on the absorption rate of a laser beam in intrusive rocks can be identified.

### Experimental setup

**Figure 2:**
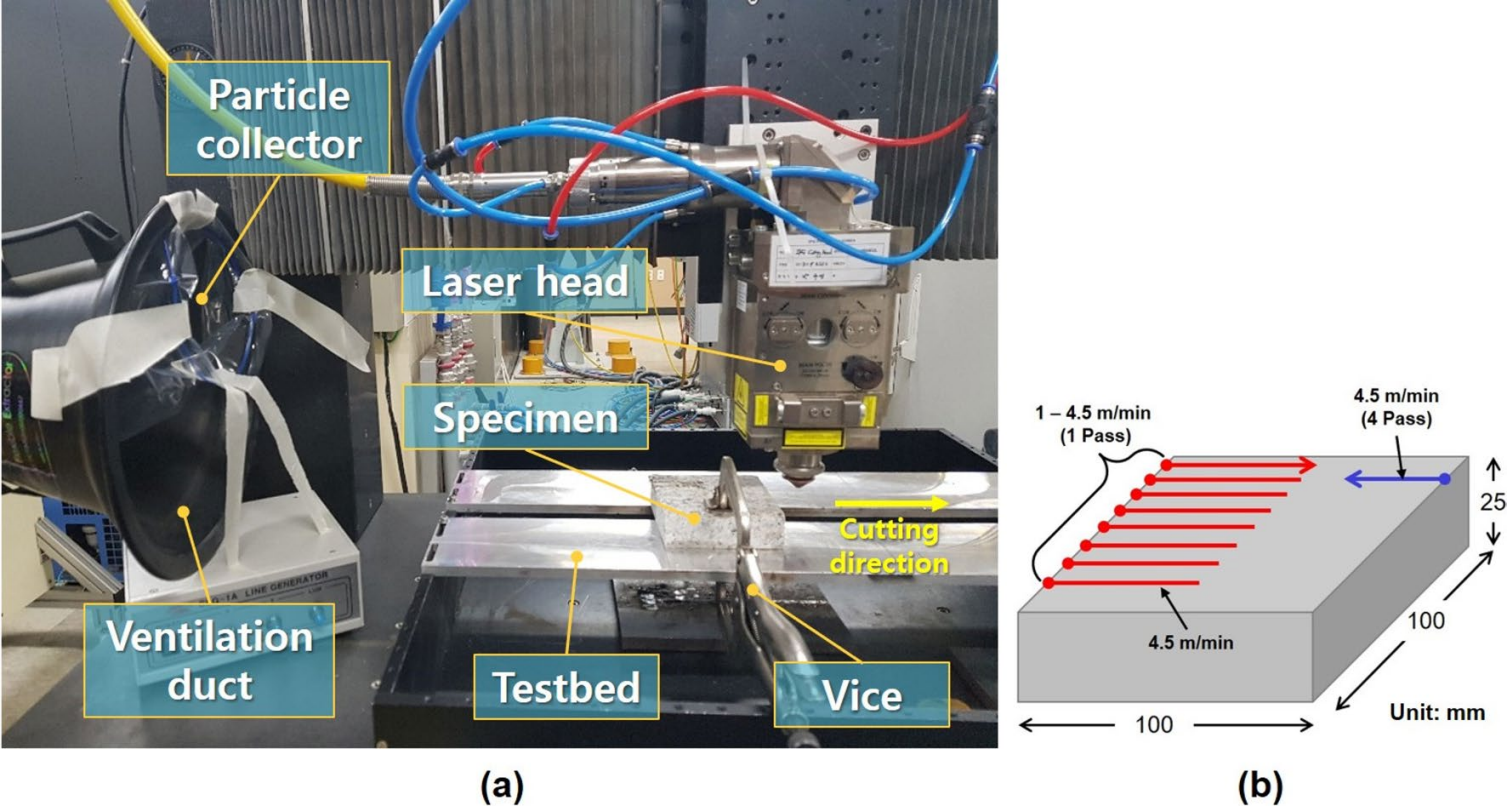
(**a**) Experimental setup, (**b**) specimen dimensions, and laser cutting paths.

The experiments were performed using a 10 kW multimode fiber laser (IPG YLS-10000 MM, IPG Photonics, Oxford, MA, USA), working at a wavelength of 1064 nm, while the laser beam was in the ${\mathrm{TEM}}_{00}$ mode. The laser beam spot size was 150 $\mathrm{\mu m}$ and its focus was positioned at 7 mm below the upper surface of the specimens in all experiments. The laser power was set at 9 kW for these experiments. The laser cutting speed, which was the only independent variable among the laser parameters, was set from 1 to 4.5 m/min in increments of 0.5 m/min. In particular, when laser cutting was at 4.5 m/min, the influence of the number of passes on the penetration depth was studied by comparing the results of 1 pass and 4 passes under the same laser parameters. The assistant gas, ${\mathrm{N}}_{2}$, was maintained at 13 bar to efficiently remove the molten materials that formed during the laser cutting process. The specimens were placed on a testbed and fixed by a vice. The interval of the testbed was set at 6.5 mm in order to facilitate the removal of material released by the assistant gas. The laser beam was irradiated vertically onto the specimens by moving the laser head. To collect the fine dust generated by the laser cutting process, a ventilation duct to which tape was attached was placed next to the laser device, as shown in Fig. 2.

Volume energy (${\mathrm{J}/\mathrm{m}}^{3}$) was generally considered for the laser cutting process. Volume energy represents an important parameter and was used to consecutively compare the irradiated laser energy per unit volume of the specimen. In addition, understanding the interaction between laser and rock through volume energy is essential in the laser cutting field. In this study, volume energy was calculated using the following equation^21^:

2 $${E}_{Volume}=\frac{P}{V\times A} \left[ \frac{J}{m^3} \right],$$

where *P* is the laser power, *V* is the laser cutting speed, and *A* is the laser beam area at the focal position. The tested volume energies in accordance with the cutting speed are summarized in Table 2.

**Table 2:** Volume energy according to the cutting speed used in the experiment.

Index	Speed [m/min]	Volume energy [$\mathrm{J}/{\mathrm{m}}^{3}$]
1	4.5	6.79E + 12
2	4	7.64E + 12
3	3.5	8.73E + 12
4	3	1.02E + 13
5	2.5	1.22E + 13
6	2	1.53E + 13
7	1.5	2.04E + 13
8	1	3.06E + 13

Subsequently, laser irradiation, kerf width, melting width, and penetration depth were observed using a Dino-lite digital microscope AM2111 (AnMo Electronics Corp., New Taipei, Taiwan). It is through kerf width and penetration depth that the amount of material removed as a result of laser irradiation can be evaluated.

## Results and discussion

### Melting and kerf widths

**Figure 3:**
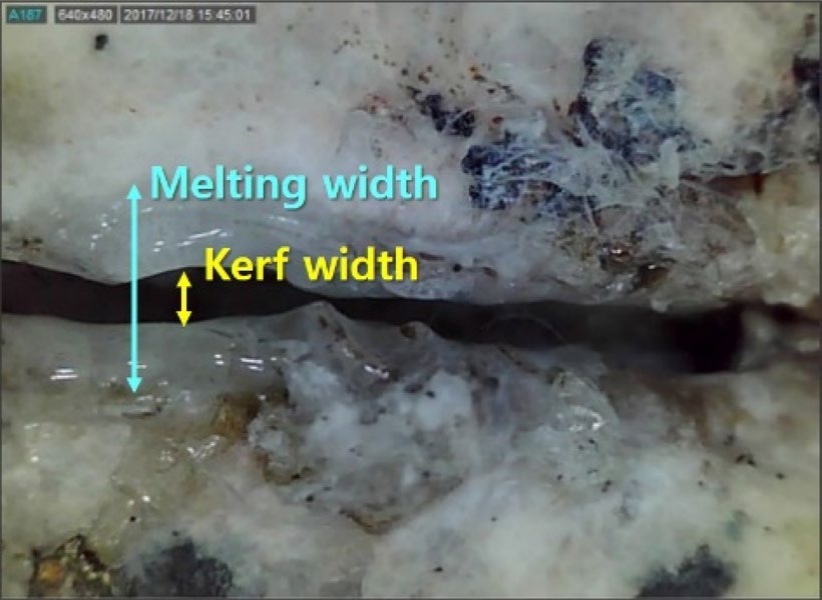
Identification of melting and kerf widths on the top surface.

**Figure 4:**
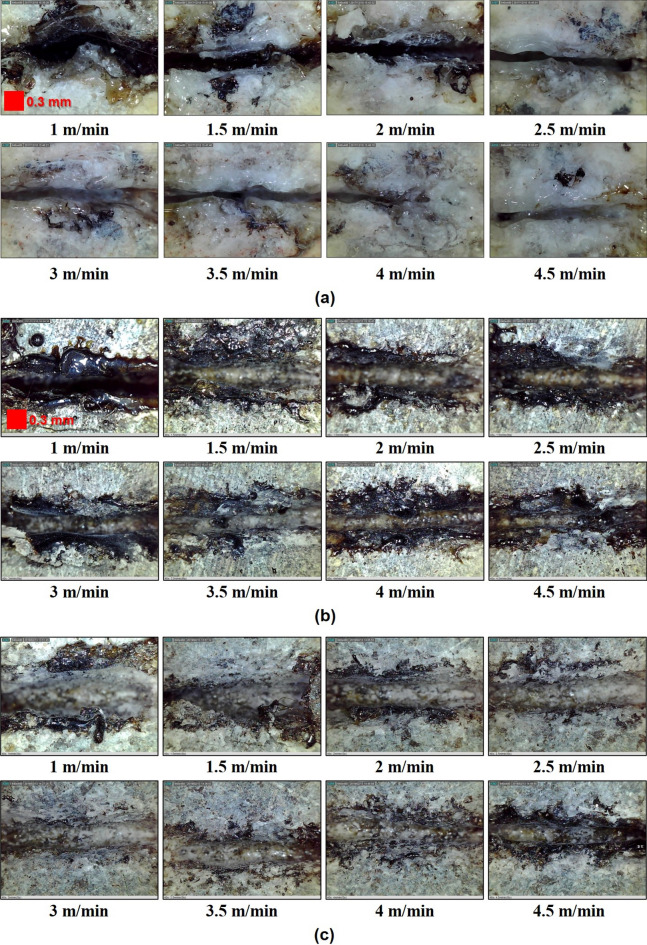
Optical images of top surfaces on (**a**) granite, (**b**) gabbro, and (**c**) diorite.

**Figure 5:**
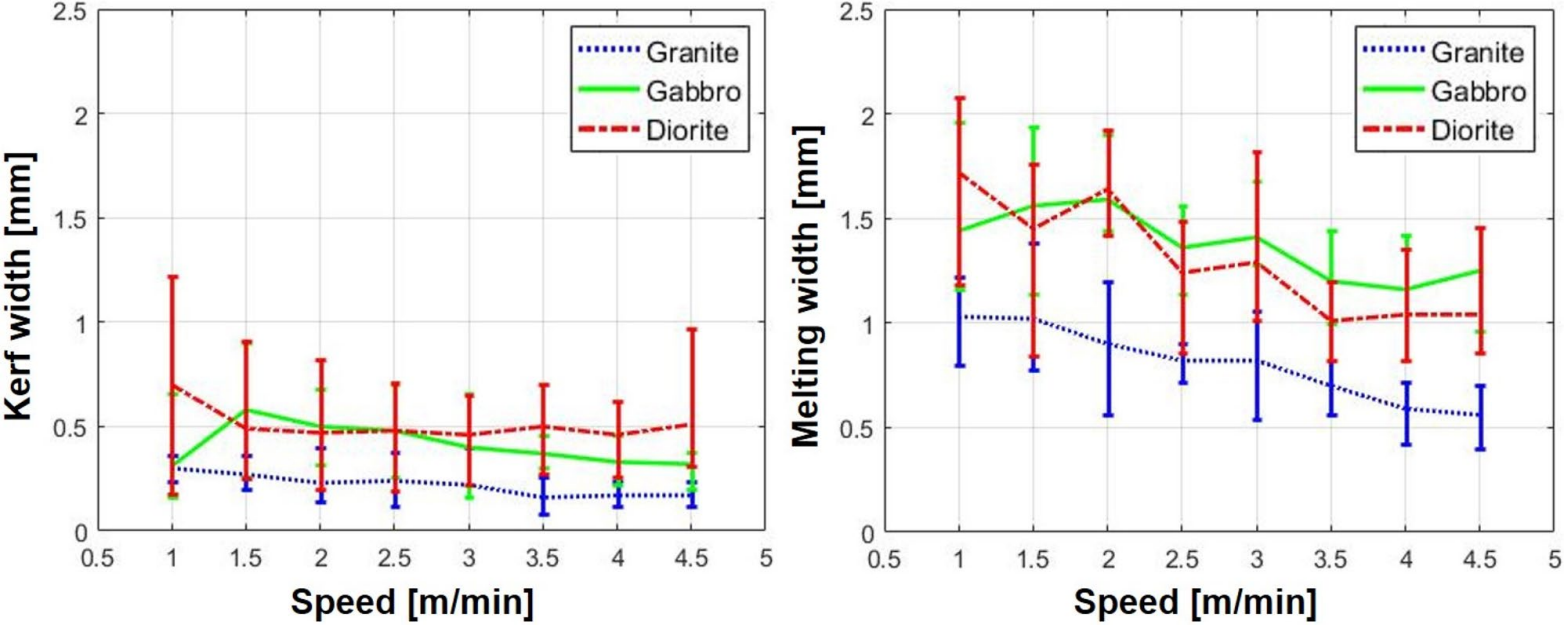
The relationship between the average kerf & melting widths and the cutting speed for each specimen.

Figure 3 shows identification of the melting and kerf widths created by the laser cutting process on the top surface of specimens. Kerf width is defined as the width of the material that is removed by the laser. Meanwhile, the melting width refers to the width of the material that is melted by laser irradiation and is then re-solidified again. Figure 4 shows the optical images of the top surface of each specimen after laser cutting. In addition, the relationship between the average cutting speed and the kerf width was indicated, as shown in Fig. 5. In granite, the kerf width gradually decreased as the laser cutting speed increased. In comparison with other specimens, granite had the smallest average kerf width of 0.3 mm and a low difference between the maximum value and the minimum value. The formation of a transparent melting zone around the cutting path was observed in granite. In gabbro, the tendency of the kerf width to decrease in accordance with the cutting speed was distinctly observed in comparison to other specimens. However, the kerf width significantly increased when the cutting speed was in the 1–1.5 m/min range. It can be assumed that the kerf width temporarily decreased as the melted material was expelled to the upper surface by high laser energy and re-solidified around the laser-irradiated zone. Diorite had the largest average kerf width of 0.59 mm. Its kerf width remained almost constant, regardless of cutting speed. Furthermore, when the cutting speed was 1 and 4.5 m/min, a large deviation in kerf width was observed.

### Penetration depth

**Figure 6:**
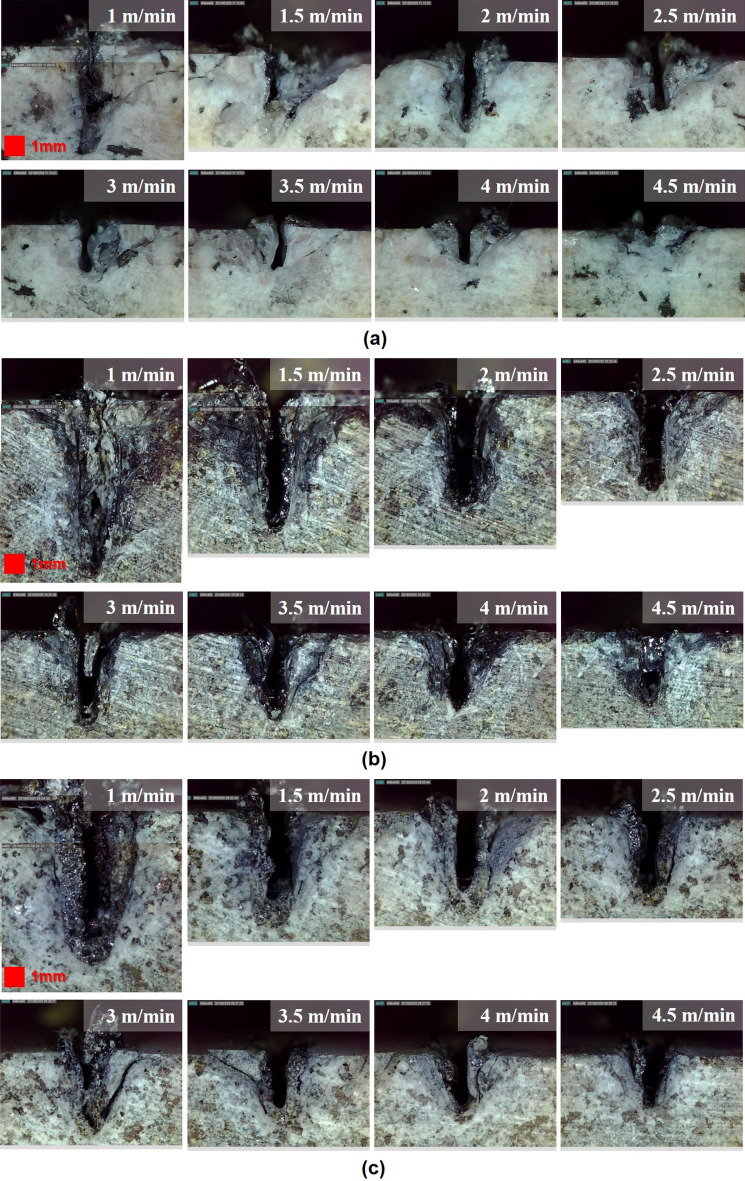
Optical images of cross-sectional views on (**a**) granite, (**b**) gabbro, and (**c**) diorite.

**Figure 7:**
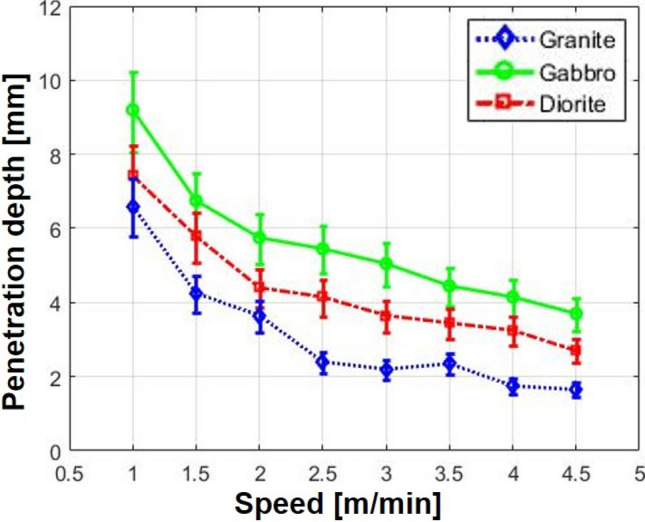
The relationship between the penetration depth and the laser cutting speed for each specimen.

Penetration depth was measured to investigate how deep the laser beam penetrated the specimen under given laser parameters. For each specimen, the penetration depth measurement was made on a cross-sectional view after mechanical cutting that was performed perpendicularly to the laser movement path. Figure 6 shows the cross-sectional view of each specimen after laser cutting. Figure 6a shows cross-sectional views of granite, where it is clear that the material from the laser-irradiated zone had been removed and that granite had the smallest HAZ of all the specimens. Unlike granite, gabbro was partially solidified and the melted materials were not completely vaporized by the laser, as can be seen in Fig. 6b. In addition, the largest HAZ—which formed around the cutting path—was observed for gabbro. In diorite, both macro- and micro-cracks occurred repeatedly in the boundary between the re-solidified zone and the HAZ. In the case of the re-solidification zone, the material was melted and then re-solidified, while the material was only affected by heat without changing phase in the HAZ. It can be assumed that once the specimen was irradiated above a certain level as a result of high laser intensity, the material started melting and then solidifying due to rapid temperature changes. To investigate the relationship between the penetration depth and the cutting speed, penetration depths were indicated as shown in Fig. 7. At the same laser cutting speed, differences in penetration depths were observed in accordance with the specimen type. It was found that penetration depth generally presents a decreasing trend as cutting speed increases. Furthermore, as shown in Fig. 1, gabbro—which had the highest absorption rate of about 88%—had the deepest penetration depth of all the specimens, while the granite—which had the lowest absorption rate—had the smallest penetration depth. In other words, a clear relationship was observed between the penetration depth and the absorption rate of intrusive rocks in terms of laser processing. When the cutting speed was 1 m/min, the penetration depths of all specimens were less than 11 mm. Based on this observation, it can be concluded that volume energy of more than 3.06E + 13 $\mathrm{J}/{\mathrm{m}}^{3}$ is required to fully remove rock specimens that have a thickness of 25 mm.

**Figure 8:**
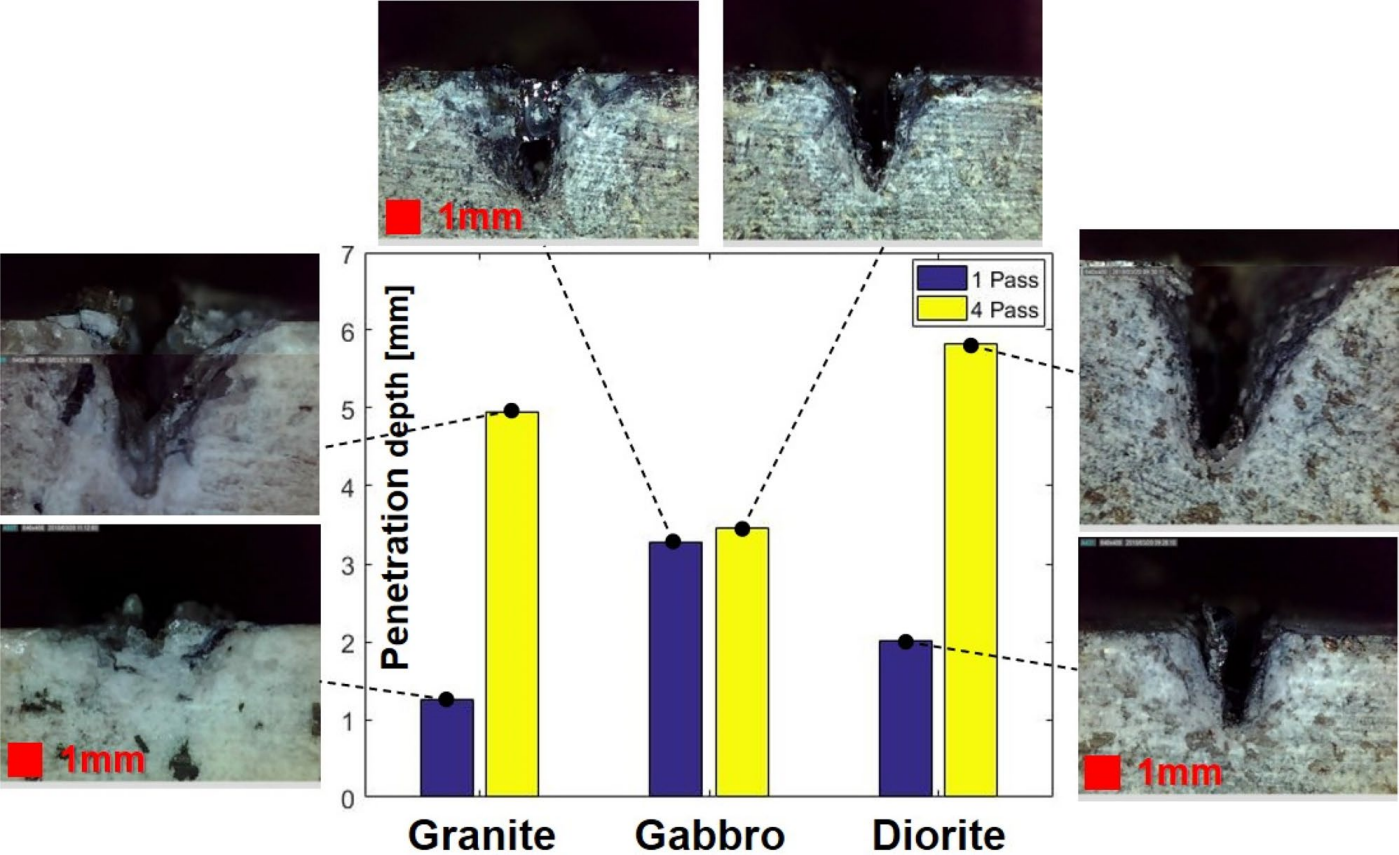
Comparison of penetration depths in accordance with the number of laser cuts.

Using the same laser cutting speed of 4.5 m/min, the number of cuts was selected by comparing the cut quality. Figure 8 shows the influence of the number of laser irradiations by comparing the penetration depth for each specimen. With 1 laser cut pass, the penetration depths of granite, gabbro, and diorite were 1.25 mm, 3.28 mm, and 2.02 mm, respectively. After 4 passes, their penetration depths were measured at 4.95 mm, 3.45, and 5.82 mm, respectively. In granite, the depth differed most significantly among the specimens, increasing by more than four times in accordance with the number of laser irradiations. The depth of diorite tripled as the number of cuts increased, and a wider kerf width was observed in comparison to that of granite. Unlike granite and diorite, no significant difference was observed in the depth of gabbro in relation to the number of cuts. It was estimated that the most laser energy was used to remove the re-solidified material in the gabbro case. In short, the materials located in the laser-irradiated zone were vaporized and the melt was simultaneously formed while the laser was initially irradiated into gabbro. After a while, the laser was unable to remove the material anymore and most of the energy was used to remove the molten material inside the penetration hole. Consequently, there was little difference in depth in relation to the number of cuts.

### Chemical analysis

#### EDX analysis for the non-processed zone

**Figure 9:**
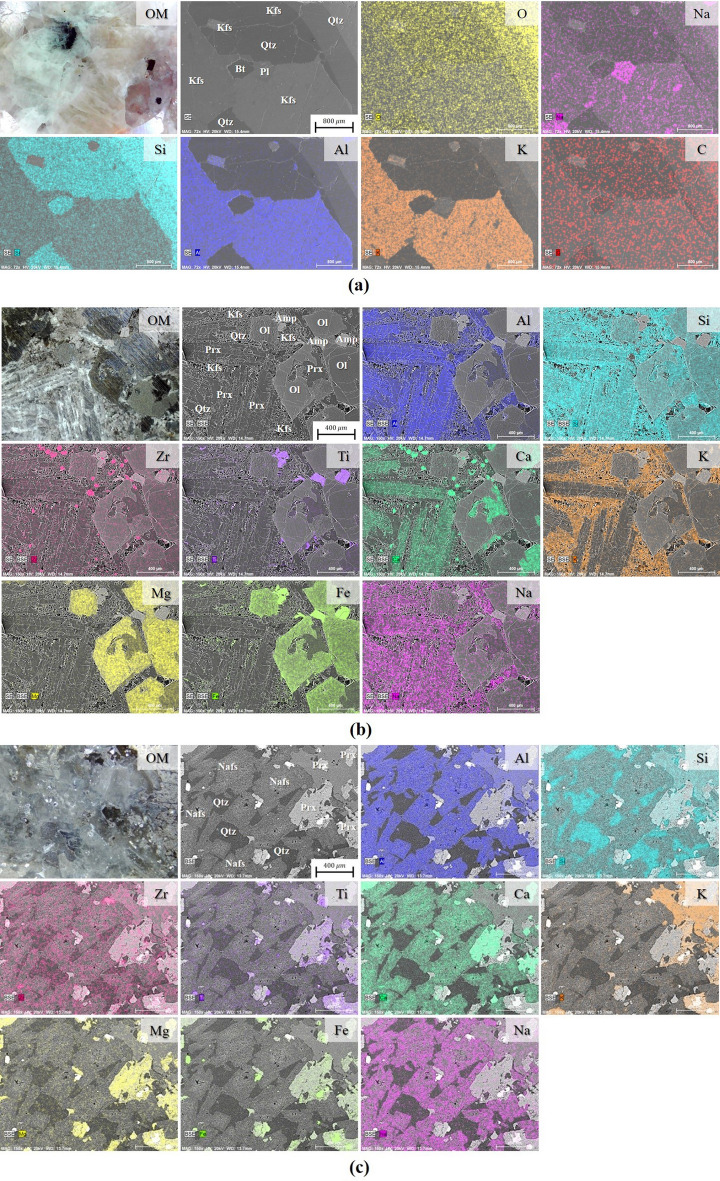
Optical microscope (OM) images and EDX element mapping for (**a**) granite, (**b**) gabbro, and (**c**) diorite. *Qtz* quartz, *Bt* biotite, *Kfs* potassium feldspar, *Pl* plagioclase, *Amp* amphibole, *Prx* pyroxene, *Ol* olivine, *Nafs* sodium feldspar.

Before observing the effect of laser cutting on the rock specimens, the microstructure and chemical composition of each rock were analyzed through the optical microscope (OM), scanning electron microscope (SEM), and energy-dispersive X-ray (EDX). In addition, the mineral types that constitute each rock were classified using EDX element mapping, as shown in Fig. 9.

Since granite contained the most silica components, silica was distributed in the entire zone—especially in quartz, where dense distributions were observed. Similarly, the distribution of many silica components was also observed in gabbro and diorite. Unlike granite, however, numerous metallic components, such as iron, magnesium, titanium, and zirconium, were observed in gabbro and diorite. In addition, granite generally had coarse-grained texture of differing sizes, which is the result of the slowly cooling magma process deep underground. In contrast, gabbro and diorite formed in small-to-medium grains that ranged from 0.420 mm to 2 mm due to their comparatively faster cooling rate in comparison to granite.

#### EDX analysis for the laser-processed zone

**Figure 10:**
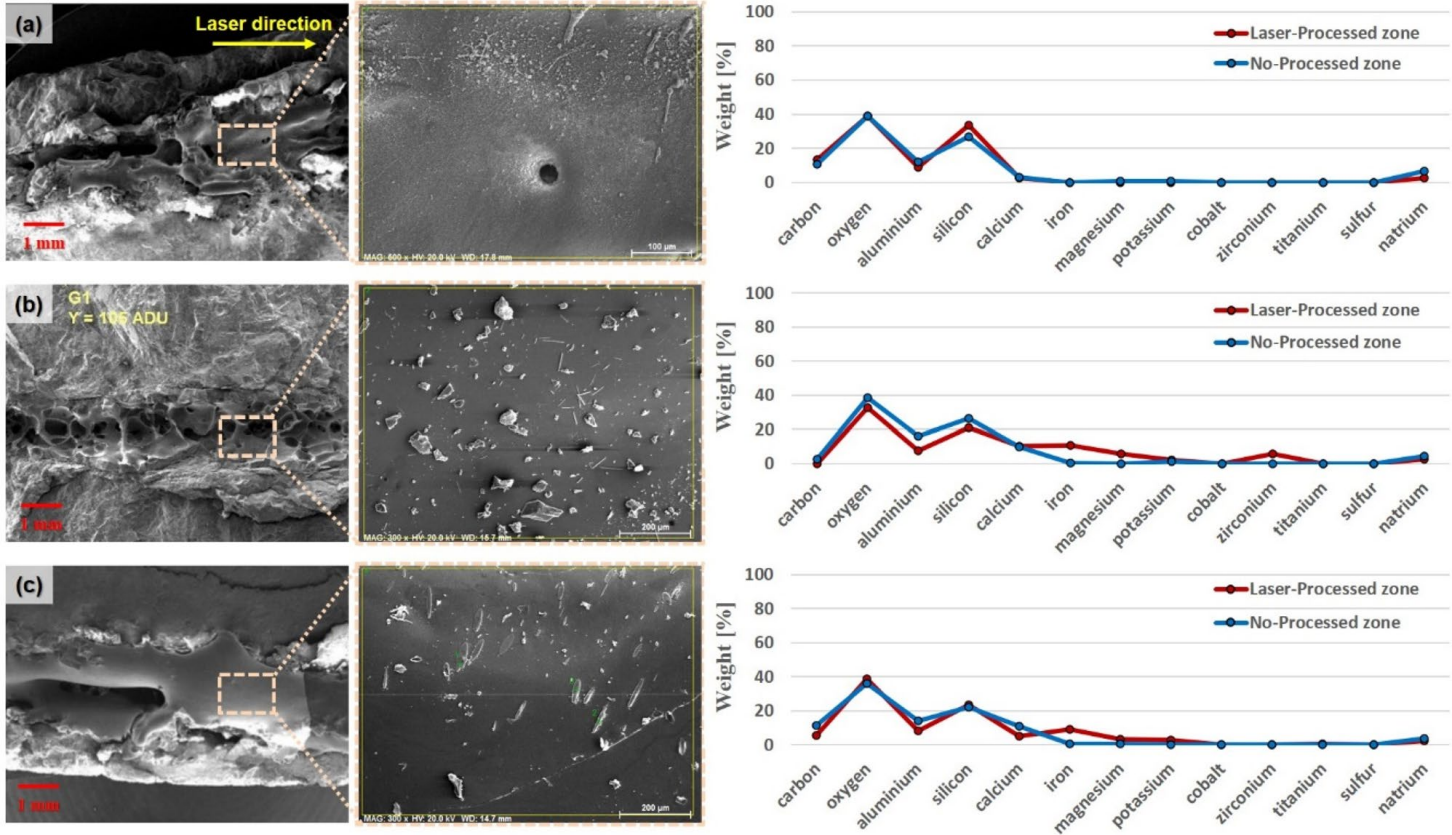
Comparison analysis between the non-processed zone and the laser-processed zone of (**a**) granite, (**b**) gabbro, and (**c**) diorite using EDX component analysis.

Following laser irradiation for each specimen, SEM observation and EDX component analysis were conducted on the laser-processed zone in order to analyze the effect of the laser on the microstructure and chemical composition of the specimens. As shown in Fig. 10, EDX component analysis was performed by designating specific positions along the laser cutting path on the top surface. A variety of minerals, such as quartz, plagioclase, and amphibole, were non-uniformly combined in all rocks. Indeed, different results could be derived during EDX analysis, depending on the measurement point. Hence, the results of the component analysis in the non-processed zone and the laser-processed zone were plotted using the average values of 10 different positions, respectively. In the case of granite, there was no considerable difference between the non-processed zone and the laser-processed zone in terms of chemical components. Although the weight percentage of silicon increased slightly in the laser-processed zone in granite, the other components were similar to those before laser cutting. In addition, many pores were observed in the laser-processed zone with an average diameter size of about 50 μm. They were formed by gas bubbles, which were generated by the thermally decomposed gases of the mineral components^22^. Meanwhile, specific chemical components, such as carbon, oxygen, aluminum, and silicon, were continuously observed in the non-processed zone, regardless of the rock type. However, metallic components were detected after laser cutting in the laser-processed zone of gabbro and diorite. This could be explained as being the result of the existence of certain minerals, such as olivine and pyroxene, and even metallic components. Thus, the molten material formed in the cutting process would also be comprised of the melt of these metallic components. However, because of the high vaporization point of these metallic components, some parts of the molten material that contained metallic components were not completely removed. For this reason, when laser processing is applied to intrusive rocks, the degree of material removal could vary depending on the types and characteristics of the minerals found in these rocks.

## Conclusion

The performance of laser cutting on intrusive rocks was investigated using a high-power fiber laser system. The experimental variables were three intrusive rock types and laser parameters, such as scanning speed and the number of laser cuts. The following conclusions were drawn from this investigation.

1. The melting width of all specimens decreased as the cutting speed increased. However, this trend was not observed in kerf width. In the case of granite, the kerf and melting widths were both significantly lower than those of gabbro and diorite.
2. The penetration depth also decreased as the cutting speed increased. When the cutting speed was 4.5 m/min, diorite had a penetration depth of about 4 mm, while the penetration depth of granite was 1.78 mm. It was identified that the depth of penetration was clearly related to the absorption rate results for each specimen.
3. Before laser cutting, the mineral types contained in each rock were classified using EDX element mapping. Metallic elements were observed in gabbro and diorite but not in granite.
4. EDX component analysis revealed that granite had almost the same components before and after laser irradiation. However, in gabbro and diorite, when examined after laser irradiation, many metallic elements were found in the laser-processed zones because their internal metal minerals had melted and mixed with other materials during the laser irradiation process.

To extend the present study, further studies aiming to identify the formation mechanisms of spattered particles are necessary in order to accurately define the principles of spattering during laser cutting. In addition, the study of spatter formation in relation to different rock types should be performed under the same laser parameters, and new LAM configurations should be developed for in-situ LAM applications on intrusive rocks.

## Data Availability

The datasets generated during and/or analyzed during the current study are available from the corresponding author on reasonable request.
